# Consensus clinical management guidelines for Alström syndrome

**DOI:** 10.1186/s13023-020-01468-8

**Published:** 2020-09-21

**Authors:** Natascia Tahani, Pietro Maffei, Hélène Dollfus, Richard Paisey, Diana Valverde, Gabriella Milan, Joan C. Han, Francesca Favaretto, Shyam C. Madathil, Charlotte Dawson, Matthew J. Armstrong, Adrian T. Warfield, Selma Düzenli, Clair A. Francomano, Meral Gunay-Aygun, Francesca Dassie, Vincent Marion, Marina Valenti, Kerry Leeson-Beevers, Ann Chivers, Richard Steeds, Timothy Barrett, Tarekegn Geberhiwot

**Affiliations:** 1 Department of Diabetes, Endocrinology and Metabolism, University Hospitals Birmingham NHS Foundation Trust, Queen Elizabeth Hospital, Birmingham, B15 2TH UK; 2 Department of Medicine (DIMED), Padua University Hospital, Padua, Italy; 3 Adult MTG3 Chair of ENDO-ERN, Azienda Ospedaliera Padova, Padua, Italy; 4 Centre de référence pour les affections rares ophtalmologiques CARGO, FSMR SENSGENE, ERN-EYE, Hôpitaux Universitaires de Strasbourg, Strasbourg, France; 5 Laboratoire de Génétique Médicale, UMRS_1112, Institut de Génétique Médicale d’Alsace, Université de Strasbourg, Strasbourg, France; 6 Diabetes Research Unit, Torbay and South Devon NHS Foundation Trust, Torquay, UK; 7 CINBIO (Centro de Investigacion Biomedica), Universidad de Vigo, Vigo, Spain; 8 Departments of Pediatrics and Physiology, College of Medicine, University of Tennessee Health Science Center and Pediatric Obesity Program, Children’s Foundation Research Institute, Le Bonheur Children’s Hospital, Memphis, TN USA; 9 Department of Respiratory Medicine, University Hospital Birmingham NHS Foundation Trust, Queen Elizabeth Hospital, Birmingham, UK; 10 Liver and Hepatobiliary Unit, University Hospitals Birmingham NHS Foundation Trust, Queen Elizabeth Hospital, Birmingham, UK; 11 Department of Cellular Pathology, University Hospitals Birmingham NHS Foundation Trust, Queen Elizabeth Hospital, Birmingham, UK; 12 Department of Medical Genetics, Abant İzzet Baysal University, Bolu, Turkey; 13 Department of Medical and Molecular Genetics, Indiana University School of Medicine, Indianapolis, IN USA; 14 Departments of Genetic Medicine and Pediatrics, Johns Hopkins University School of Medicine, Baltimore, MD USA; 15 Italian Association Alström Syndrome, Padua, Italy; 16 ENDO-ERN ePAG representative in MTG3, Padua, Italy; 17 Alström Syndrome UK, Torquay, Devon UK; 18 Department of Cardiology, University Hospitals Birmingham NHS Foundation Trust, Queen Elizabeth Hospital, Birmingham, UK; 19 Department of Endocrinology and Diabetes, Birmingham Women’s and Children’s Hospital NHS Foundation Trust, Birmingham, UK; 20 Institute of Metabolism and System Research, University of Birmingham, Birmingham, UK

**Keywords:** Alström syndrome, Guidelines, Rare disease, Blindness, Deafness, Cardiomyopathy, Insulin resistance, Obesity, Non-alcoholic fatty liver disease

## Abstract

Alström Syndrome (ALMS) is an ultra-rare multisystem genetic disorder caused by autosomal recessive variants in the *ALMS1* gene, which is located on chromosome 2p13. ALMS is a multisystem, progressive disease characterised by visual disturbance, hearing impairment, cardiomyopathy, childhood obesity, extreme insulin resistance, accelerated non-alcoholic fatty liver disease (NAFLD), renal dysfunction, respiratory disease, endocrine and urologic disorders. Clinical symptoms first appear in infancy with great variability in age of onset and severity. ALMS has an estimated incidence of 1 case per 1,000,000 live births and ethnically or geographically isolated populations have a higher-than-average frequency. The rarity and complexity of the syndrome and the lack of expertise can lead to delayed diagnosis, misdiagnosis and inadequate care. Multidisciplinary and multiprofessional teams of experts are essential for the management of patients with ALMS, as early diagnosis and intervention can slow the progression of multi-organ dysfunctions and improve patient quality of life.

These guidelines are intended to define standard of care for patients suspected or diagnosed with ALMS of any age. All information contained in this document has originated from a systematic review of the literature and the experiences of the authors in their care of patients with ALMS. The Appraisal of Guidelines for Research & Evaluation (AGREE II) system was adopted for the development of the guidelines and for defining the related levels of evidence and strengths of recommendations.

These guidelines are addressed to: a) specialist centres, other hospital-based medical teams and staffs involved with the care of ALMS patients, b) family physicians and other primary caregivers and c) patients and their families.

## Background

Alström Syndrome (ALMS; OMIM #203800) is an ultra-rare multisystem genetic disorder caused by pathogenic variants of *ALMS1* gene. This syndrome was first described by Carl-Henry Alström in 1959 as a progressive retinal degeneration, obesity, neuronal hearing loss and insulin resistance [1]. Clinical features, time of onset and severity can vary greatly among and even within families bearing identical genetic alterations. The disease is relentlessly progressive in nature which can result in premature death.

The rarity of this disorder, the complexity of the syndrome and the scarcity of expertise can translate into misdiagnosis, delayed diagnosis, and barriers to adequate care. This results in inadequate or inappropriate treatment, loss of confidence in the healthcare system, and patient disempowerment. To date, there is no disease specific therapy for ALMS and the mainstay of management involves multidisciplinary and multiprofessional teams of experts as early diagnosis and intervention can slow the progression of multi-organ dysfunctions and improve the longevity and quality of life of patients. There are no national or international management guidelines/ standard operating procedures to guide how best to care for patients with ALMS. The European Alström Syndrome society (ASEU) (www.alstromeurope.org) has initiated the development of a comprehensive disease management guideline to provide a resource for the multidisciplinary team, and to support patients, caregivers and their primary care professionals on the current diagnosis, treatment, monitoring and outcome measures for patients with ALMS. This document represents general guidelines, which in the opinion of the authors can inform care providers about the needs of patients with ALMS in order to provide equitable and improved care. It defines standard of care for ALMS patients, fosters shared care arrangements between expert centres and family physicians, and empowers patients. The guidelines refer to the management of patients suspected or diagnosed with ALMS of any age. These guidelines should be of value to: a) specialist centres, other hospital-based medical teams and other staffs involved with the care of ALMS patients, b) family physicians and other primary caregivers and c) patients and their families.

## Methods

These guidelines have been developed by International expert physicians, geneticists, allied healthcare professionals and patient support groups with a common aim to support equitable care by standardising the standard of care for all patients with ALMS. The Guidelines Development Group (GDG) consisted of expert representatives from a range of professional groups including paediatric and adult endocrinologists, metabolic specialists, cardiologists, ophthalmologists, Ear, Nose and Throat (ENT) specialists, respiratory specialists, pathologists, geneticists, basic scientists, specialist metabolic dieticians, physiotherapists, psychologists, specialist nurses and patient support group representatives. The GDG Committee agreed the remit of the guidelines and selected a list of guidelines topics for development.

A systematic literature review on ALMS of the last 45 years until October 2019 was carried out using Medline, MedLink, Embase and the Cochrane Library. Relevant published papers considered by the GDG members as important were included. The initial search identified 220 reference abstracts, of which 179 were accepted as relevant after the first screen. References related to a single topic were pulled together and the GDG was divided into subgroups aimed to critically appraise references devoted to a specific topic (Genetics and Diagnosis, Hearing and Sight, Cardio-respiratory system, Endocrine and Metabolism, Gastro-intestinal and Genito-urinary tract, Psychosocial aspects, and overall management and follow up). The committee discussed by teleconference in August 2019 and met in October 2019 in Birmingham, UK. They also corresponded by email on a regular basis throughout the duration of the guidelines development. During the workshop, the GDG adopted the second version of the Appraisal of Guidelines for Research & Evaluation (AGREE II) system as methodological reference in order to meet the guideline development standards. Relevant papers were evaluated by members of the GDG before the evidence was considered. Section leaders individually assessed the literature and wrote a short document describing the study findings and related recommendations. All GDG members discussed the draft documents. Evidence levels were classified in accordance with the Grading of Recommendations, Assessment, Development and Evaluations (GRADE) methodology and level of evidence were graded from A to C (Table 1). In addition, for the adoption of recommendations, we formed a panel of experts representative of the specialists caring for ALMS patients and used the Delphi method for the development of our guidelines. The guidelines will be published in an open access journal and made freely accessible through the ALMS society website. These guidelines will be revised every 3–5 years to reflect new data pertaining to future research findings, new therapies and the development of diagnostic methods. The development of these guidelines was made without external financial support from industries involved in the manufacturing of therapies for ALMS. Competing interests of members of the guideline development group have been recorded in writing and addressed.

**Table 1:** Evidence levels and strength of recommendations

Item	Definition
Level of evidence	
*A. High-quality evidence*	Further research is unlikely to change our confidence in the estimate of effect. Consistent evidence from the Randomised Controlled Trials (RCTs) without important limitations or exceptionally strong evidence from observational studies.
*B. Moderate-quality evidence*	Further research is likely to have an important impact on our confidence in the estimate of effect and may change the estimate. Evidence from RCTs with important limitations (inconsistent results, methodologic flaws, indirect or imprecise), or very strong evidence from observational studies.
*C. Low-quality evidence*	Further research is very likely to have an important impact on our confidence in the estimate of effect and is likely to change the estimate. Evidence for at least one critical outcome from observational studies, case series, or from RCTs with serious flaws, or indirect evidence, or expert’s consensus.
Strength of recommendation	
*1. Strong recommendation*	Recommendation can apply to most patients in most circumstances.
*2. Weak recommendation*	The best course of action may differ depending on circumstances or patient or society values. Other alternatives may be equally reasonable.

## Definition and epidemiology

### How do you define Alström syndrome (ALMS)?

***Statement # 1:*** *ALMS is a progressive and multi-systemic disorder caused by autosomal recessive variants in the ALMS1 gene. The disease is characterized by visual and hearing impairments, cardiomyopathy, renal dysfunction, extreme insulin resistance (IR) and its related complications. Clinical symptoms first appear in infancy with great variability in age of onset and severity.*

- *Level of evidence: A*
- *Strength of recommendation: 1*
- *Experts’ opinion: completely agree (88%), mostly agree (12%), partially agree (0%), mostly disagree (0%) and completely disagree (0%)*

### How common is ALMS?

***Statement # 2****: Alström syndrome is a pan-ethnic ultra-rare condition, with an estimated incidence of 1 case per 1,000,000 live births. Ethnically or geographically isolated populations have a higher-than-average frequency of ALMS.*

- *Level of evidence: C*
- *Strength of recommendation: 2*
- *Experts’ opinion: completely agree (75%), mostly agree (19%), partially agree (6%), mostly disagree (0%) and completely disagree (0%)*

The prevalence of ALMS is difficult to estimate as some individuals with attenuated forms of this syndrome may be underdiagnosed [2]. There is a higher frequency of some specific *ALMS1* pathogenic variants in certain ethnic populations, for example, c.10534_10535ins in French Acadians, and c.10775delC in up to 20% of patients of English descent [3, 4]. Based on our collective national Alström service experience in Europe there are at least 263 cases known to us including 89 in the UK, 64 in France, 60 in Turkey, 39 in Italy and 15 in Spain.

## Molecular and genetic basis of the disease

***Statement # 3:*** *ALMS is a monogenic disorder caused by homozygous or compound heterozygous variants in the ALMS1 gene, which is located on chromosome 2p13. Of the 268 pathogenic variants identified so far, 96% are nonsense or frameshift changes (insertions and deletions).*

- *Level of evidence: A*
- *Strength of recommendation: 1*
- *Experts’ opinion: completely agree (81%), mostly agree (19%), partially agree (0%), mostly disagree (0%) and completely disagree (0%).*

The syndrome was first described by Carl Henry Alström in 1959 in three cousins [1]. Goldstein & Fialkow, in 1973 [5], described a new Alström family and reviewed the clinical phenotype of the three families described until then. They proposed an autosomal recessive pattern of inheritance. *ALMS1* gene is located on chromosome 2 (region 2p13.1) [6–8] and contains 23 coding exons [4, 9] that comprises 224 kilobase (kb). The ALMS1 protein is of unknown function, is widely expressed in human and mouse tissues [4, 9, 10], and localizes to centrosomes and the base of cilia [11]. To date, over 268 pathogenic variants have been involved in ALMS [4, 9, 12–15] of which 96% are nonsense or frameshift changes (insertions and deletions) that could produce truncated, non-functional proteins if translated [14]. In addition, splice-site mutations [16], deletions [17], one Alu transposon insertion [18] and one balance translocation [9] have been described. Among the 24 missense variants in the ALMS1 database, 11 (45.8%) are predicted to be pathogenic, four (16.7%) are likely pathogenic, four (16.7%) are benign or likely benign, and five (21%) are variants of uncertain significance [15]. Taking into account the different cohorts reported, most of deleterious variants are clustered in exons 8 (6.1 kb), 10 (1.9 kb) and 16 (1.2 kb), which are considered mutational hotspots as they comprise 85–97% of the total mutational load for *ALMS1* [14, 19].

## Clinical presentation

### What are the principal manifestations raising the suspicion of ALMS?

***Statement # 4:*** *ALMS is a multisystem, progressive disease characterised by visual disturbance, hearing impairment, cardiomyopathy, childhood obesity, extreme insulin resistance, accelerated non-alcoholic fatty liver disease (NAFLD), renal dysfunction, respiratory disease, endocrine and urologic disorders. First symptoms usually occur during the first year of life either with visual disturbance and/or heart failure and clinical findings evolve as affected individuals age.*

- *Level of evidence: A*
- *Strength of recommendation: 1*
- *Experts’ opinion: completely agree (94%), mostly agree (6%), partially agree (0%), mostly disagree (0%) and completely disagree (0%)*

#### Eyesight and hearing

##### Visual impairment

***Statement # 5:*** *Retinal dystrophy is a major and consistent manifestation (incidence 100%) of the disease leading to visual impairment that is often severe and can lead to blindness before the age of 20. Visual impairment is often one of the first symptoms or even the very first noticeable manifestation appearing between a few weeks and 6 months of age.*

- *Level of evidence: A*
- *Strength of recommendation: 1*
- *Experts’ opinion: completely agree (88%), mostly agree (12%), partially agree (0%), mostly disagree (0%) and completely disagree (0%)*

Patients typically present with early onset retinal dystrophy due to the degeneration of photoreceptor cells (cones and rods). At birth, patients do not usually have manifestations, but within a few weeks signs of visual impairment occur. The parents usually observe nystagmus (“wobbling eyes”), early onset photophobia (avoidance of light) and very poor visual contact. Visual symptoms occur early in the first months but some cases are later in onset with a slower evolution [20, 21]. The first referral to an ophthalmologist occurs usually before the age of one. The rate of visual loss is severe from the outset and yearly examination will show rapid deterioration. The visual impairment is often profound and reaches legal blindness most often during the first decade with teenagers and young adults registered as blind and, almost all patients having lost vision before the second decade.

***Statement # 6:*** *Ophthalmic investigations are necessary to evaluate the progressive visual consequences and support the diagnosis. The initial assessment will include standard ophthalmic evaluation followed by retinal imaging and functional testing (performed according to the age of patient and level of participation).*

- *Level of evidence: A*
- *Strength of recommendation: 1*
- *Experts’ opinion: completely agree (93%), mostly agree (7%), partially agree (0%), mostly disagree (0%) and completely disagree (0%)*

The diagnosis of retinal dystrophy is based on the clinical presentation, visual functional evaluation and ocular imaging. The standard evaluation will define the visual state of the child according to the age of the child, categorize the oculomotor presentation (horizontal and/or pendular nystagmus; strabismus with often exotropia), evaluate the refraction (usually under routine cycloplegia with drops that inhibits accommodation to measure accurately the power of glasses), and perform indirect ophthalmoscope to visualize the fundus. Main tests to assess visual impairment in ALMS are summarised in Table 2.

**Table 2:** Main tests for visual assessment in ALMS

**Refraction status (mandatory)**	It often shows a hyperopia (ranging from + 3.5 up to + 12) and astigmatism. Myopia is not common. Full corrective glasses must be prescribed as long as some vision is still present.
**Visual acuity (mandatory)**	Visual acuity is often very low early on (less than 1/10 with Decimal test or 6/60 with 6 m Snellen test) evolving to residual light perception around the age of 10. However, some cases have shown a later and milder onset with higher values of vision.
**Slit lamp examination (mandatory)**	The fundus examination varies according to age and among individuals independently from the genetic status or the extra ocular status. Chronologically the fundus examination can reveal: near normal appearance, attenuated vessels, hypo-pigmented appearance to the retina with increased visibility of the choroidal vessels, macula pigmentary changes, pale optic discs and /or papillary drüsen often observed. Later global pigment mottling and local clumps can appear whereas classical “bone spicules” are seldom reported. Crystalline deposits have been observed [21].
**Electroretinography (ERG)** **(mandatory)**	The usual characterization is an early onset severe cone-rod dystrophy and confirmation must be made by a full-field ERG. ERG shows very early and usually profound alteration of the light adapted photopic responses due to severe alteration of the cones. The follow up highlights a rapid evolution with diminishing dark-adapted rod responses. Before the end of the first decade, the ERG become un recordable and shows extinguished cone and rod responses (absent responses using any type of stimuli).
**Goldman visual field (Optional)**	Goldman or Humphrey Visual fields are often unrecordable. Exceptionally more or less pronounced visual field constriction have been reported and even very exceptionally with some preservation [20, 21].
**Optic Coherence Tomography (OCT)** **(optional)**	The OCT findings are scarcely reported [21–24], because the recording is difficult to achieve especially in young children with nystagmus, photophobia and need for sedation. Thinning of the macula and immature retina with persistence of the foveal inner retinal layer were reported [22, 23] but this was challenged by further reports that showed mild central macular changes in the first decade followed by loss of photoreceptors and pigment epithelium that correlated with the low vision [21, 24]. The OCT images in adults show general atrophy of both inner and outer retinal layers. Thinning of the outer nuclear layer and the outer plexiform layer outside the fovea is a constant finding [21].
**Fundus Auto Fluorescence (FAF)** **(optional)**	FAF is seldom reported with hyper autofluorescent parafoveal ring in two patients and small areas of hypo and hyperfluorescent zones outside or inside the arcades [21].

##### Deafness

***Statement # 7:*** *Hearing impairment is the second most common manifestation of ALMS and characterised by progressive bilateral sensorineural hearing loss. No abnormality is detected during new born screening, but deafness has been diagnosed as early as the age of 1 and 70% before the age of 10 with lifetime risk of 100%.*

- *Level of evidence: B*
- *Strength of recommendation: 1*
- *Experts’ opinion: completely agree (63%), mostly agree (37%), partially agree (0%), mostly disagree*
  *(0%) and completely disagree (0%)*

Hearing problems are common manifestations of ALMS that appear during the course of the disease, often during childhood, as a progressive sensorineural deafness. High frequency ranges are the first affected in both ears. ENT assessment including otologic examination and audiological evaluation of both ears is necessary to confirm the diagnosis and support the patient. Absent oto-acoustic emissions with intact speech discrimination and normal auditory brain stem responses points to a cochlear origin [25–31]. The pathogenesis is thought to be due to the alteration of outer hair cells of the cochlea that occurs after birth [32, 33]. In addition to the neurosensorial dysfunction, a conductive component can occur. The average age of progression of hearing loss is 10-15db/decade [33].

#### Cardiovascular disease

##### How do patients with ALMS present with cardiovascular disease?

***Statement # 8:*** *Almost two-thirds of individuals with ALMS develop congestive heart failure (CHF) at some stage in their lives, as a result of infantile, juvenile or adult onset cardiomyopathy. Approximately 40% infants with ALMS suffer from a transient, severe cardiomyopathy between 3 weeks and 4 months of age. In those who survive, there is a risk of recurrence and in those without infantile cardiomyopathy,* de novo *disease develops in approximately 1 out of 5 patients.*

- *Level of evidence: B*
- *Strength of recommendation: 1*
- *Experts’ opinion: completely agree (67%), mostly agree (33%), partially agree (0%), mostly disagree (0%) and completely disagree (0%)*

Onset, progression and outcome of cardiomyopathy in ALMS is variable. The first clinical presentation of the syndrome is either with nystagmus or infantile onset cardiomyopathy in 42% within the first 4 months of life [25]. In the absence of routine genetic testing of those presenting with infantile cardiomyopathy, diagnosis is likely to be under-estimated due to premature mortality in a significant proportion. Of those who recover, approximately 1 in 5 then go on to have either recurrent disease or present de novo with symptoms of congestive cardiac failure. The characteristic feature of juvenile or adult onset cardiomyopathy is diffuse interstitial and coarse replacement myocardial fibrosis on post-mortem or using non-invasive cardiovascular magnetic resonance imaging [34]. Symptoms on presentation include reduced exercise capacity, breathlessness on exertion, orthopnoea, ankle swelling, nocturnal cough and wheeze. Reduced exercise capacity and breathlessness are relatively sensitive symptoms but lack specificity for heart failure, due to the frequency of co-existing issues in ALMS, including physical de-conditioning, obesity and reversible airways disease. Physical signs are difficult to interpret and lack sensitivity and specificity, as many with ALMS have no abnormal signs, particularly when the degree of heart failure is mild. Tachycardia lacks sensitivity and blood pressure is often normal or patients are treated for hypertension [35]. Likewise, oedema is more common in those who are immobile or obese, while crepitations in the lung fields may be common due to poor ventilation or to the development of fibrosis rather than heart failure. Signs such as elevated jugular venous pressure and displacement of the apex beat are more specific but are much harder to elicit and have poor reproducibility [36].

##### How is cardiovascular disease diagnosed in Alström syndrome?

***Statement # 9:*** *Although an abnormal electrocardiogram may increase the likelihood of cardiovascular disease in individuals with ALMS, and elevated plasma concentrations of natriuretic peptides can be used as an initial diagnostic test, transthoracic echocardiography is the most useful and widely available test for diagnosis, estimating prognosis and monitoring response to treatment.*

- *Level of evidence: C*
- *Strength of recommendation: 1*
- *Experts’ opinion: completely agree (75%), mostly agree (17%), partially agree (8%), mostly disagree (0%) and completely disagree (0%)*

Plasma concentrations of natriuretic peptides can be a useful initial test for diagnosis of CHF in patients with ALMS, since those with normal levels are unlikely to have CHF. Their use is mainly in excluding disease, since there are other causes for elevated levels, including advancing age > 50 years, pulmonary hypertension and renal dysfunction, while obesity may lower levels [37].

Abnormalities on the 12-lead electrocardiogram occur in a third of patients with ALMS but changes are non-specific and include axis deviation, criteria for left ventricular hypertrophy, poor R wave progression and non-specific ST changes [35]. It was initially suggested that transthoracic echocardiography commonly identified abnormalities in structure and function in infants and children with ALMS, with a high frequency of left ventricular dilatation, globally impaired left ventricular ejection fraction without regional wall motion abnormalities and reduced right ventricular function [38]. Subsequent studies using echocardiography and cardiovascular magnetic resonance imaging in larger populations of subjects have indicated that overt reduction in ejection fraction may be present in approximately a third but that ventricular size is commonly normal [35]. Using gold standard cardiovascular magnetic resonance imaging, left and right ventricular size is commonly normal in adults, and mass is within normal range with normal wall thickness [34]. Of interest, sub-clinical left ventricular (LV) dysfunction has been documented on advanced image analysis with 2D feature tracking and reduction in global longitudinal strain, and there remains a question whether this may be more common in those who recovered from infantile cardiomyopathy [39]. It is not known whether reduction in global longitudinal strain is predictive of adverse events, as in the general adult population**.**

##### What is the role of myocardial fibrosis in Alström syndrome?

***Statement # 10****: There is evidence on post-mortem and on non-invasive cardiovascular imaging of coarse replacement and diffuse interstitial fibrosis in the heart. It is not known whether this is direct consequence of the abnormal ALMS1 protein or a result of the metabolic derangements that are associated with ALMS.*

- *Level of evidence: B*
- *Strength of recommendation: 1*
- *Experts’ opinion: completely agree (74%), mostly agree (13%), partially agree (13%), mostly disagree (0%) and completely disagree (0%)*

Post-mortem evidence in the largest series of cases presented to date demonstrated myocardial fibrosis in the hearts of 3 out of 4 patients with cardiomyopathy and mild fibrosis in the heart of one patient who had no history of cardiovascular disease [25]. Non-invasive multiparametric cardiovascular magnetic resonance imaging demonstrated coarse replacement fibrosis in 5 of 7 adult patients with Alström syndrome, with a typical pattern of late gadolinium enhancement within the mid wall of the left ventricle [40]. While late gadolinium enhancement quantifies coarse replacement fibrosis, it does not detect diffuse interstitial myocardial fibrosis, which has been identified in most adult patients using T1 mapping and extracellular volume quantification [41]. There is some evidence that fibrosis is progressive, but data are unclear whether this structural change is causative or a marker of the development of cardiomyopathy. Data have suggested that the presence of fibrosis may be reflected in sub-clinical changes in function measured by strain [34]. There are currently no effective treatments known to reverse myocardial fibrosis in ALMS**.** Fibroblast primary cultures obtained from patients with ALMS up-regulated the expression and production of collagens displaying a constitutively activated myofibroblast phenotype even if they do not derive from a fibrotic lesion [42]. It is not clearly demonstrated if the multi-organ fibrosis could be a direct consequence of ALMS1 dysfunction or a result of secondary metabolic alterations.

##### Do patients with ALMS develop coronary artery disease?

***Statement # 11****: Occult coronary disease is present in one third of adult patients with Alström syndrome before 50 years of age but symptomatic disease does not occur in children and is rare in adults.*

- *Level of evidence: C*
- *Strength of recommendation: 1*
- *Experts’ opinion: completely agree (73%), mostly agree (18%), partially agree (9%), mostly disagree (0%) and completely disagree (0%)*

Metabolic disturbances are cardinal features of ALMS and begin in childhood, with effects including rapid weight gain, disproportionate IR, type 2 diabetes mellitus (T2DM) and dyslipidaemia. The high prevalence of risk factors, particularly when combined with the difficulties in following an active lifestyle imposed by visual and hearing impairment, increase the likelihood of coronary artery disease. Despite this, reported cases of symptomatic coronary artery disease are uncommon [43] although there is post-mortem evidence on histology of asymptomatic myocardial infarction [34]. On this basis, it seems reasonable to follow a strategy of aggressive risk reduction in patients, noting that previous reports of impaired LV function and cardiomyopathy have not excluded coronary artery disease in a structured manner.

#### Endocrine and metabolic functions

##### Empty Sella, hypothyroidism, short stature

***Statements # 12:*** *Total or partial empty sella may present in ALMS and it might represent the morphological underpinning of endocrine dysfunctions of the pituitary gland. Hypothyroidism and growth hormone deficiency are common findings. The majority of children have a rapid growth, with height above the 50th percentile before puberty but final adult height frequently below the 5th percentile.*

- *Level of evidence: B*
- *Strength of recommendation: 1*
- *Experts’ opinion: completely agree (33%), mostly agree (50%), partially agree (17%), mostly disagree (0%) and completely disagree (0%).*

Hypothyroidism (range 11–36%) and sub-clinical hypothyroidism have been reported in patients with ALMS [19, 25, 44, 45]. Hypothyroidism may be primary or secondary and it is related to autoimmunity in 20% of cases [45].

Up to 50% normal weight patients with ALMS have an inadequate GH reserve and may be functionally GH deficient [19, 44, 46]. Few patients have been treated with rhGH [46, 47]. After 1 year of rhGH treatment, body fat mass, fat infiltration in the liver, and serum lipid profiles had all decreased; insulin sensitivity and acanthosis nigricans improved [47]. Short stature may be at least partially influenced by impairment of the IGF-1 axis [45, 48] or pituitary fibrosis [44]. Patients with ALMS and controls have similar arm span/height ratios and sitting/standing height ratios [45]. A skeletal age of 1–3 years in advance of chronological age has been reported in children [19, 25, 44]. Thoracic and lumbar scoliosis, kyphosis, or lordosis is present in 68% of patients with ALMS [25].

In a cohort of 38 patients a diagnosis of central adrenal insufficiency was found in a single case [45]. No alterations of prolactin have been reported to date. Central diabetes insipidus was reported in a single case [49].

##### Male hypogonadism and female hyperandrogenism

***Statement # 13****: Male hypogonadism and female hyperandrogenism are common findings in ALMS. Microphallus, undescended testes, hypospadias, small testes and gynecomastia can be present in men. Women can show hirsutism, alopecia, oligomenorrhea or amenorrhea, polycystic ovaries, high testosterone levels, abnormal breast development, precocious puberty and endometriosis.*

- *Level of evidence: B*
- *Strength of recommendation: 1*
- *Experts’ opinion: completely agree (71%), mostly agree (29%), partially agree (0%), mostly disagree (0%) and completely disagree (0%).*

Hypergonadotropic and hypogonadotropic hypogonadism are common in males [19, 25, 44, 45]. Histopathologic findings showed testicular atrophy with obliterating fibrosis of seminiferous tubules [25]. Males are unlikely to be fertile. Erectile dysfunction and sexual drive have not been thoroughly investigated. Testosterone replacement therapy should be offered as per the national guidelines.

Women present normal external genitalia, uterus, and fallopian tubes; however, menstruation is often scant, sporadic, or irregular. Histopathological studies showed extensive fibrosis of the ovary and devoid of oocytes, follicles, and corpora lutea [25]. A relatively high frequency of ovarian cysts is reported, which may be associated with obesity and hyperinsulinemia. Female fertility is unlikely and no patients have reproduced so far. Metformin, estrogens, progestins, or their combination might be helpful to normalise menstrual irregularities.

##### Childhood obesity

***Statement # 14****: Childhood obesity occurs in most of patients with ALMS. Although birth weight is within the reference range, rapid weight gain can be observed within 2 to 36 months. Waist circumference, Body Mass Index (BMI) and percent fat mass tend to decrease with age after adulthood.*

- *Level of evidence: B*
- *Strength of recommendation: 1*
- *Experts’ opinion: completely agree (43%), mostly agree (50%), partially agree (7%), mostly disagree (0%) and completely disagree (0%)*

Obesity is one of the cardinal features of ALMS [16, 50, 51]. ALMS is characterized by fat accumulation in subcutaneous instead of visceral regions [52]. Adipose tissue (AT) histology showed disordered pro-inflammatory and fibrotic gene expression profiles [53]. Adipocytes of subcutaneous and visceral AT were larger than controls in mouse model [54] and human specimens of patients with ALMS [53].

Leptin levels are elevated and correlate with body weight [48]. However, mild elevations in leptin levels normalized to BMI, suggest leptin resistance [55]. Resting energy expenditure is comparable with controls but the hyperphagia score may be higher, suggesting that higher intake rather than the lower metabolic rate is probably the primary driver for obesity in ALMS [45]. In the mouse model fat Aussie (foz/foz), hyperphagia was observed before weight gain [56].

##### Insulin resistance, type 2 diabetes and dyslipidaemia

***Statement # 15****: Almost all individuals with ALMS are prone to insulin resistance (IR). The severity and degree of complications of IR increase with age and body habitus and yet disproportionate to BMI. Patients with ALMS have a 10-fold greater prevalence of metabolic syndrome; most patients during their lifetime develop type II diabetes and moderate to severe dyslipidaemia.*

- *Level of evidence: A*
- *Strength of recommendation: 1*
- *Experts’ opinion: completely agree (93%), mostly agree (0%), partially agree (7%), mostly disagree (0%) and completely disagree (0%)*

In a large cohort of patients with ALMS, hyperinsulinemia usually developed between ages 18 months and 4 years [25]. Type 2 diabetes mellitus (T2DM) can be diagnosed as early as age 5 years, with a median age at onset of 16 years [25]. Eighty-two percent of patients older than 16 years were diabetic [25]. The severity of IR is more than 10 times that of BMI-matched patients with obesity [45].

In the ALMS1 GT/GT and foz/foz, mouse models, hyperinsulinemia develops early and pancreatic islets show beta cell proliferation, thus suggesting that both IR and increased insulin secretion might contribute to glucose intolerance [10, 56]. Using zebrafish models, loss of *Alms1*, resulted in a significant decrease in β-cell production and under prolonged exposure to high glucose conditions, β-cells were unable to continually expand as a result of decreased proliferation and increased cell death [57, 58]. The knockdown of *Alms1* resulted in excess secreted insulin and cellular transport, especially in insulin-related pathways, prior to the onset of adipogenesis. These findings support a role for *Alms1* in regulating secretion and membrane depolarization in β-cells [58]. In addition, a role for ALMS1 in glucose homeostasis and GLUT4 translocation in adipose tissue of ALMS1 GT/GT mouse model was shown [54]. In patients with ALMS, ectopic fat deposition in the liver and potentially skeletal muscle could play a key role in the IR development [45].

Most patients have moderate to severe hypertriglyceridemia [range 200-1000 mg/dL (2.26–11.29 mmol/L)], with normal total cholesterol and low HDL cholesterol levels [25, 45, 59, 60]. ALMS patients with triglyceride levels greater than 1000 mg/dL (11.3 mmol/L) are prone to develop acute pancreatitis [25]. In addition, low levels of apolipoprotein A1 [60] and HDL [45] were reported. Unexpectedly, LDL-cholesterol was lower in patients with ALMS compared with BMI-matched controls [45].

##### Non-alcoholic fatty liver disease

***Statement # 16****: Non-alcoholic fatty liver disease (NAFLD) is common in ALMS with a high tendency to progress to non-alcoholic steatohepatitis (NASH) and fibrosis disproportionate to age, BMI and duration of T2DM. Plasma concentration of liver enzymes is often elevated starting in early childhood. In some affected individuals, liver disease progresses to cirrhosis and hepatic failure in the second to third decade. Portal hypertension associated with splenomegaly, oesophageal varices, ascites, and hepatic encephalopathy may occur.*

- *Level of evidence: B*
- *Strength of recommendation: 1*
- *Experts’ opinion: completely agree (93%), mostly agree (7%), partially agree (0%), mostly disagree (0%) and completely disagree (0%)*

Advanced cases of NAFLD were reported in the UK adult cohort of 30 patients with ALMS and T2DM [53]. Advanced cirrhosis of the liver can occur in teenage years and in the absence of severe obesity, diabetes or alcohol excess. Conversely some obese severely insulin resistant and diabetic ALMS persons have been found to have normal livers at autopsy. Liver biopsies and post-mortem examination have revealed varying degrees of NASH, liver fibrosis, cirrhosis, and more nonspecific chronic active hepatitis with lymphocytic infiltration and patchy necrosis [25, 61]. Early stages of NASH (inflammation, hepatocyte ballooning, steatosis) can remit and relapse with significant improvements in weight loss (in the obese cases), exercise tolerance, IR and blood sugar [62]. More research is needed to determine whether advanced cirrhosis as opposed to steatohepatitis is driven primarily by direct genetic effects on the liver rather than secondary effects of the syndrome such as degree of IR.

#### Respiratory involvement

##### Do ALMS patients have respiratory problems?

***Statement # 17:*** *There is a higher prevalence of upper and lower respiratory tract infection in ALMS. Children with ALMS have an increased incidence of respiratory infections, which seems to reduce during adulthood. Conventional Lung Function testing is often difficult due to dual sensory loss and problems with coordinating of deep inspiration and forced expiration. When performed, many cases show a restrictive pattern, often with a maintained transfer coefficient of the lung for carbon monoxide (KCO).*

- *Level of evidence: B*
- *Strength of recommendation: 1*
- *Experts’ opinion: completely agree (69%), mostly agree (31%), partially agree (0%), mostly disagree (0%) and completely disagree (0%)*

Recurrent otitis media is very common at all ages and tympanostomy tubes are often placed in children with chronic otitis media to prevent recurrence and complications. Boerwinkle et al. reported episodes of bronchitis, pneumonia and sinusitis in more than half of 38 patients with Alström syndrome [63].

Restrictive lung disease is frequently described and it can be due to extrapulmonary restriction from obesity or less commonly kyphoscoliosis, sometimes in combination with alveolar and interstitial fibrosis. In case of severe and progressive scoliosis, surgery may be considered. In less severe cases or if the risks of surgery are considered too high, braces could be an alternative.

Histopathological studies have shown early morphological changes indicating an inflammatory process in the small airways [27] and post mortem studies have described alveolar and interstitial fibrosis.

There is a reported increased susceptibility of sudden and severe hypoxaemia during or following anaesthesia or surgery [64]. The underlying mechanisms for severe hypoxia is unknown but could be related to cytokine storm triggered by host immune response.

#### Renal and urological complications

##### Renal disease

***Statement # 18****: Chronic kidney disease is common, slowly progressive, and highly variable. Onset can be in mid-childhood through adulthood. End-stage renal disease can occur as early as the mid- to late teens.*

- *Level of evidence: B*
- *Strength of recommendation: 1*
- *Experts’ opinion: completely agree (81%), mostly agree (19%), partially agree (0%), mostly disagree (0%) and completely disagree (0%)*

Renal ultrasonography and Magnetic Resonance Imaging (MRI) may reveal abnormalities. The most common ultrasonography finding is renal parenchymal hyperechogenicity often limited to the medulla. Renal cysts are identified in a small number of patients [65, 66]. Renal biopsy often shows interstitial fibrosis, glomerular hyalinosis, and tubular atrophy but absence of histopathologic features of diabetic or reflux nephropathy [25, 65]. In addition, glomerular function in Alström syndrome does not show significant association with T2DM, hyperlipidemia, cardiomyopathy, or hypertension, suggesting that kidney disease may be the primary manifestation of the syndrome. Diabetes, hypertension and hyperuricemia may have an additive effect on the progression of renal disease. Microalbuminuria is common but not overt proteinuria despite the presence of T2DM. Obstructive uropathy is rare. It is reported in the literature a case of successful kidney transplant because of chronic renal failure in a 42-year old man with ALMS [67].

##### Dysuria

***Statement # 19****: Up to 50% of patients with ALMS have reported dysuria and or long periods without voiding urine. These symptoms have been attributed to detrusor-urethral dys-synergia (lack of coordination of bladder and urethral muscle activity). More severe problems appear to occur in females in their late teens. Lower abdominal and perineal pain is common and may relate to bladder spasm.*

- *Level of evidence: B*
- *Strength of recommendation: 1*
- *Experts’ opinion: completely agree (69%), mostly agree (23%), partially agree (8%), mostly disagree (0%) and completely disagree (0%)*

There has been no systematic study of urological function in ALMS. Manifestations include urinary frequency, incontinence, and symptoms associated with recurrent infections [31, 68]. More severe manifestations are rare (< 2%) and include worsening urinary incontinence or retention; these symptoms may alternate.

A few case reports describe severe detrusor urethral dysfunction in female patients with ALMS. The detrusor spasm interfered with all aspects of daily living and was partially or not responsive to antispasmodics or self-catheterisation. In all cases, urinary diversion procedure with ileal conduit was performed and symptoms relieved after surgery.

#### Gastrointestinal dysfunction

***Statement # 20****: General gastrointestinal disturbances such as epigastric pain and gastro- esophageal reflux disease are common. Rarely, spontaneous caecal volvulus can occur.*

- *Level of evidence: C*
- *Strength of recommendation: 1*
- *Experts’ opinion: completely agree (64%), mostly agree (9%), partially agree (18%), mostly disagree (9%) and completely disagree (0%)*

Recurrent symptoms of epigastric pain, nausea, regurgitation of food and heartburn were found in 19% of 178 patients with ALMS compared with 8% of children without the syndrome [69]. There have been no reports of systematic investigation of gastro-oesophageal reflux in the syndrome. Two genetically confirmed cases have been investigated and treated for severe reflux which had interfered with quality of life [15]. One proceeded to successful gastric plication.

Caecal volvulus has been reported in a sibling pair with genetically confirmed ALMS [70]. No other cases of small or large bowel volvulus have been reported. It is possible that a co-inherited anatomical deformity of the bowel underlay the volvulus rather than the presence of ALMS.

#### Developmental and neurological observations

***Statement # 21:*** *Developmental milestones can be delayed in ALMS individuals, most commonly in gross and fine motor skills. Learning disability and mixed receptive-expressive language delays are also reported. Cognitive impairment (IQ < 70) is very rare. The ages at which children first sit, walk, and organise phrases of at least 2 words can be used as psychomotor developmental milestones during childhood.*

- *Level of evidence: C*
- *Strength of recommendation: 1*
- *Experts’ opinion: completely agree (54%), mostly agree (33%), partially agree (13%), mostly disagree (0%) and completely disagree (0%).*

The most comprehensive analysis of psychosocial outcomes in the syndrome was led by Marshall et al. and reported in the review defining the phenotype in 182 cases of the syndrome [25]. Mentation and neurobehavioral traits were estimated on the level of school and social performance and interviews with parents. Intelligence tests included the Wechsler Adult Intelligence Scale–Revised, the Bayley Scales of Infant Development, Birth to Three Test of Language and Learning Development, Behaviour Assessment System for Children, and the Haptic Intelligence Scale for the Adult Blind. Developmental milestones were delayed in 39 males and 35 females (46%). Autistic-spectrum behavioural abnormalities were common.

##### Do patients with Alström syndrome develop neurological disease?

***Statement # 22:*** *Neurological manifestations such as seizure and hyporeflexia can occur up to 20% patients. Subtle brain volume and intensity changes were noted in neuroimaging assessments.*

- *Level of evidence: C*
- *Strength of recommendation: 1*
- *Experts’ opinion: completely agree (64%), mostly agree (22%), partially agree (14%), mostly disagree (0%) and completely disagree (0%).*

Hyporeflexia was observed in 20% of patients (age range, 9 months to 41 years). 12% of these patients had frequent absence seizures. Birth complications included neonatal hypoxia and hypotonia. Excessive startle, partial unilateral paralysis, unexplained joint or muscle pain and muscle dystonia were also reported. 16% had generalized sleep disturbances (age range, 3–41 years) [25]. Other smaller studies have shown wide heterogeneity in theory of mind performance. For example, with many patients with ALMS performing as well as controls, but those with earlier visual loss showing impaired responses to abstract questioning [71].

One study has compared patients with ALMS with controls using conventional MRI, Voxel-Based Morphometry (VBM) and Diffusion Tensor Imaging (DTI). In contrast with patients under investigation for epilepsy, matched for age and gender, there were early vascular-like lesions, grey and white matter atrophy, mostly involving the posterior regions, and diffuse supratentorial white matter derangement. These ALMS related cerebral structural change might underlie behavioural and developmental problems in a minority of patients [72].

##### Psychosocial aspects

***Statement # 23:*** *The effects of complete visual loss or realisation can result in severe anxiety and depression often in late adolescence. Anxiety and depression, learning challenges and barriers to engagement in society are influenced by age at diagnosis, the cultural setting in which the child with ALMS grows up, life limiting complications, and the ability to respond to the challenge of visual loss.*

- *Level of evidence: C*
- *Strength of recommendation: 2*
- *Experts’ opinion: completely agree (80%), mostly agree (20%), partially agree (0%), mostly disagree (0%) and completely disagree (0%).*

Psychological and sociological consequences of the syndrome have been reviewed by questionnaires and interviews with families. Progress can be compromised by: lack of aids to low vision, Braille and computer voice mail; development of deafness and lack of good audiology support; the reaction especially in adolescence to an understanding of the severity of the condition particularly blindness. If early education, treatment and prevention of complications and integration in society can be offered from infancy, there is a much greater likelihood of good mental and physical wellbeing.

The immense relief at finding the cause of visual impairment and obesity is coupled with consternation and fear at the realization of the serious, incurable, and progressive nature of ALMS. Severe neuronal deafness can have devastating effects of withdrawal and depression if adequate digital hearing aids are not available in a time sensitive manner. Moreover, episodes of screaming, aggression, and withdrawal from engagement with family have been described in an adolescent with sudden complete loss of vision.

## Diagnosis and differential diagnosis of Alström syndrome

The symptoms and signs of ALMS vary with the age at diagnosis. Visual impairment is the earliest and most consistent observation in all patients starting from few weeks of life. There are numerous conditions raising the suspicion of ALMS and other age appropriate diseases should also be excluded.

***Statement # 24****: In the first few years of life, history of visual impairments and/or cardiomyopathy/heart failure should raise the possibility of ALMS. The differential diagnosis includes other causes of retinal dystrophies such as Leber’s Congenital Amaurosis, Achromatopsia, Bardet-Biedl or Early Onset Cone rod dystrophy.*

- *Level of evidence: B*
- *Strength of recommendation: 1*
- *Experts’ opinion: completely agree (75%), mostly agree (25%), partially agree (0%), mostly disagree (0%) and completely disagree (0%).*

Night blindness is not a common feature in ALMS as opposed to other forms of inherited syndromic retinal dystrophies, mostly nystagmus and photophobia are the leading signs. The clinical context (obesity, cardiomyopathy) and finally molecular testing will categorise definitively the precise type of syndromic retinal dystrophy as ALMS. Since clinical diagnosis could overlap with other conditions, targeted gene panels that includes *ALMS1* gene should be employed, in order to exclude other clinical diagnosis in one molecular test. Targeted panel for ciliopathies, retinal diseases, cardiomyopathy or even obesity should include *ALMS1* gene. As children age, the syndromic features of ALMS become more apparent with metabolic, hearing, cardiac and renal related manifestations coming to prominence.

***Statement # 25:*** *Once ALMS is suspected based on age appropriate syndromic features, diagnosis can be confirmed by identification of biallelic pathogenic variants in ALMS1 gene.*

- *Level of evidence: A*
- *Strength of recommendation: 1*
- *Experts’ opinion: completely agree (75%), mostly agree (0%), partially agree (25%), mostly disagree (0%) and completely disagree (0%).*

There is no biochemical, histological or imaging test to confirm the diagnosis of ALMS and molecular genetic study is the only option in confirming the diagnosis. To overcome the challenges of identifying both biallelic pathogenic variants in ALMS1 gene, diagnostic criteria based on cardinal clinical features and family history were proposed [25]. However, given the overlap of clinical features with other syndromic ciliopathy/retinopathy and the advance in molecular genetic study attempt should be made to identify two pathogenic variants to confirm or refute the diagnosis of ALMS.

The classical molecular approach is to sequence the hotspot exons (8, 10 and 16) and if no mutation is detected, the entire coding sequence is analysed. Nowadays, the implementation of the new sequencing technologies is replacing this classical approach. Next generation sequencing-based gene panels for retinal diseases, syndromic ciliopathies and syndromic obesity or whole-exome sequencing (WES) of the proband could be performed, for the entire exon content. Checking the coverage and length of reads of the reaction is compulsory in order to produce a good detection rate. If no, or only one, pathogenic variant is detected, other strategies should be taken. Multi ligation probe assay (MLPA) could potentially be used to detect large deletion or insertions in regions not included in the exonic analysis. Whole genomic sequencing (WGS) provides a full spectrum of genetic variations, not only point mutations and small insertions/deletions but also larger copy number variations and variants that is located in noncoding regions. Although the vast majority of mutations in *ALMS1* gene have been described once, several populations showed a founder effect like the UK or Turkish population [3].

## Management

ALMS is not yet curable but is a treatable condition. Optimal disease management requires a multi-disciplinary, multi-professional team (MDT) based in a specialist centre, closely liaising with community care providers (Table 3). The mainstay of therapy is addressing the existing/impending complications and symptom management. Van Groenendael et al. [77] undertook a cross-sectional study of UK patients with ALMS and their carers. They showed that patients who have access to a highly specialised clinical service, reported high levels of satisfaction in their care. Patient treatment compliance and clinic attendance was better in a multidisciplinary clinic compared to the usual standard of care. This was associated with a significantly improved quality of life. In particular it is important to underline the role of Patients’ Associations in giving information and contacts to resolve medical and technical problems and to support patients and families creating opportunities to enjoy life.

**Table 3:** Multidisciplinary assessment of patients with ALMS

Discipline	Features of ALMS for which this discipline may be of assistance	Initial Assessment	Follow up	Reference
Primary care physician	Assist with general medical care; coordinate specialists; provide support for family	First referral at the time of diagnosis.	6 monthly or les as per clinical need	Expert opinion
Geneticists/ clinical scientists	Diagnosis of ALMS and exclusion of other disorders in the differential diagnosis; provide counselling for families as to recurrence risk and option for prenatal diagnosis if desired.	First referral prenatal or during childhood. Initial assessment: detection of two *ALMS1* biallelic variations in the proband. Then, assessment of family segregation to establish the parental alleles.	As per request or clinical need	[15, 25, 31]
Ophthalmologists	Blindness, nystagmus, photophobia; retinal dystrophy.	First referral usually before the age of one. Initial assessment includes: standard ophthalmic evaluation, retinal imaging and functional testing (performed according to the age of patient and level of participation).	Annually	[73]
ENT specialist	Progressive bilateral sensorineural hearing loss.	First referral usually during childhood. ENT assessment includes otologic examination and audiologic evaluation of both ears.	Annually	[33]
Cardiologist	Infantile, juvenile or adult onset cardiomyopathy; hypertension; coronary artery disease; heart failure.	First referral possibly before the age of one. Initial assessment: natriuretic peptides, ECG, transthoracic echocardiography (TTE). In older children and adult, include CMR.	ECG - yearly TTE – yearly or as per clinical need CMR every 3–5 years	[35, 40]
Pulmonologist	Assess for pulmonary fibrosis; restrictive lung disease; pulmonary hypertension.	First referral usually during adulthood. Initial assessment includes Conventional Pulmonary Function test (cPFT) and Chest X-ray. HRCT Thorax in cases of unexplained cough or breathlessness.	cPFT – yearly HRCT Thorax – as per clinical need	[63, 74]
Endocrinologist/ Metabolic specialist	Assess and treatment of metabolic complications (obesity, insulin-resistance, type II diabetes, non-alcoholic fatty liver disease, dyslipidaemia) and endocrine disorders (hypothyroidism, GH deficiency, male hypogonadism, female hypoandrogenism).	First referral during childhood Initial assessment: 1. Anthropometric measurements 2. Thyroid Function Test (TFT), Pituitary and sexual hormones 3. Blood glucose, HbA1c and lipid profile.	Every 6–12 months in children, then yearly Yearly Every 6–12 months or as per clinical need	[13, 25, 45, 48]
Gastroenterologist/hepatologist	Assess for liver fibrosis/cirrhosis and the complications (portal hypertension, hepatocellular cancer, liver failure).	First referral: from childhood to adulthood Initial assessment: Liver function tests, platelet count, liver ultrasound, transient elastography and ELF test. Upper gastrointestinal endoscopy (EGD) in case of cirrhosis	Yearly or as per clinical need Liver ultrasound yearly or as per clinical need.	[53]
Nephrologists	Assess for progressive renal dysfunction, Chronic Kidney Disease.	First referral: from mid-childhood to adulthood Initial assessment: Kidney function test (including microalbuminuria) and renal ultrasonography	Yearly or as per clinical need	[65]
Neurologist	Assess of developmental milestones, learning disability and mixed receptive-expressive language delays; seizure and hyporeflexia.	First referral during childhood. Initial assessment: Neurological examination, level of school and social performance, interviews with parents and intelligence tests.	As per clinical need	[27, 72]
Anaesthesiologist	Assess for anaesthetic risk.	First referral as per clinical need.	As per clinical need	[74]
Clinical psychology/ behavioural therapy team	Anxiety, isolation and depression. Support to personal and group activity.	First referral: from mid-childhood to adulthood Initial assessment includes:	Yearly or as per clinical need	[71] Expert opinion
Physioterapist	Aerobic physical exercise.	First referral: from childhood to adulthood, as per clinical need. Initial assessment: static and dynamic physical examination.	Yearly or as per clinical need.	Expert opinion
Dietician	Lifestyle modification counselling, personalised diet and weight management.	First referral during childhood.	Every 6–12 months or as per clinical need	[75]
Speech and language therapist	Assess of the sensorial impairment including speech perception, speech recognition and sound localisation and distance evaluation	First referral during childhood Consider implication of sensorial impairment in the communication, social interactions, emotional wellbeing, mobility, assistive technology, habitation and rehabilitation potential.	Yearly or as required	[76]
Social worker	Support of patients and families living with disabilities, who require enhanced resources in the community	First referral: from childhood to adulthood	As required	Expert opinion
Patients’ Association	Support to patients and their families Facilitate clinic and research	First referral at the time of diagnosis	As required	[77]

***Statement # 26:*** *Patients with ALMS exhibit progressive multisystem disease manifestations and benefit from multidisciplinary follow up from physicians and allied health care professionals with experience in caring for ALMS patients in a one stop clinic set up. Wherever possible, individuals diagnosed with ALMS should be referred to a centre with expertise in the care of this condition.*

- *Level of evidence: B*
- *Strength of recommendation: 1*
- *Experts’ opinion: completely agree (94%), mostly agree (6%), partially agree (0%), mostly disagree (0%) and completely disagree (0%).*

Depending on the country’s health care service setup, level of expertise and patients need, a MDT can be formed to enable ALMS patients to receive a collaborative management plan from a wide range of experts in an integrated manner. The MDT should have a Care Coordinator assigned to the patient serving as a primary contact point to the rest of the team. Specialists in the different disciplines have to discuss and integrate information and management as much as possible. Treatment goals are defined and agreed with the patient and their family and regularly reviewed as required. The MDT should aim to work together in a “one stop” clinic set up in integrating investigations and clinic appointments. The MDT will also benefit from a post clinic meeting in forming a consolidating care plan in a coordinated manner.

### What optimal therapy should be considered for a patient with ALMS?

The following functional assessments should take place at the time of diagnosis or symptom onset and at regular intervals thereafter for optimal symptom control and functional capacity.

#### Growth and development, eyesight and hearing

##### Growth and development

***Statement # 27:*** *The growth of children with ALMS (height, weight, head & waist circumference) should be assessed at regular intervals (not less than 6 monthly) using age appropriate instruments by their primary health care provider. In addition, adult patients should undergo a careful assessment and change in their anthropometric measurements including BMI, body fat composition and waist circumference.*

- *Level of evidence: B*
- *Strength of recommendation: 1*
- *Experts’ opinion: completely agree (75%), mostly agree (19%), partially agree (6%), mostly disagree (0%) and completely disagree (0%).*

##### Eyesight

***Statement # 28:*** *Individuals with ALMS should undergo a comprehensive eye assessment at the time of diagnosis and thereafter on regular interval. Visual loss occurs with age in all individuals with ALMS and early planning should be in place in the use of Braille, computing and adaptive living skills while vision is still present.*

- *Level of evidence: B*
- *Strength of recommendation: 1*
- *Experts’ opinion: completely agree (94%), mostly agree (6%), partially agree (0%), mostly disagree (0%) and completely disagree (0%).*

A yearly ophthalmic examination by an experienced ophthalmologist is recommended and then by a centre that has expertise in retinal dystrophies, dedicated to monitor the ophthalmic status including [31, 77]: best visual acuity or low vision evaluation; refraction; slit lamp examination to detect cataracts; fundus examination; OCT FAF and ERG if possible (Table 2).

In caring for their visual impairments treatment goals and challenges should be discussed with the patient at the outset and as the disease progress. Symptomatic therapies include:

1. Full refraction glasses should be prescribed as long as some retinal function is detected and residual vision is present. Red-orange tinted filters have proven to be very useful to mitigate photophobia.
2. Detection of cataract (mostly subcapsular) can lead to a surgical procedure (lens removal with implantation) usually in the second or third decade. This can improve visual symptoms (especially photophobia) although the benefits on vision may be limited and tempered by the state of the underlying retinal degeneration.
3. Rehabilitation for gradually deteriorating vision must be carried out by an expert team with early intervention with the use of low vision aids, magnifiers, mobility aids (white cane, guide dogs) as well as Braille learning. In addition, Smartphone, tablets and the use of multiple apps are currently available for use by severely visually impaired persons. Voice activated technologies are of major help. The goal is to achieve autonomy and independence.

To date, there is no specific gene therapy that has reached the stage of clinical trial (due to the size of *ALMS1* gene). Retinal implants have been advocated in late stage retinal dystrophies but we are not aware of any patient with ALMS having benefited from these devices.

##### Hearing

***Statement # 29:*** *People with ALMS should undergo a hearing assessment at the time of diagnosis and thereafter annually. When appropriate, patients should be offered hearing devices to improve general communication.*

- *Level of evidence: B*
- *Strength of recommendation: 1*
- *Experts’ opinion: completely agree (88%), mostly agree (12%), partially agree (0%), mostly disagree (0%) and completely disagree (0%).*

Hearing problems are a common manifestation that starts during childhood as a progressive sensorial deafness [25, 26]. In addition to the neurosensorial dysfunction, more than 42% of the children present with otitis media (glue ear) and have to undergo myringotomy. Chronic recurrent otitis media is commonly reported even in young adults [63]. This can add a conductive component to the deafness. Patient should be followed by age appropriate air bone conduction pure tone thresholds, speech thresholds, ability to word recognition with diagnostic audiometers, tympanometry, otologic examination and microscopic examination of both ears.

Hearings aids are very useful and have to be regularly monitored and adapted. Cochlear implantation has been reported and should be considered as it may have an excellent functional outcome [73, 78, 79].

#### The heart, cardiovascular and respiratory systems in ALMS

##### Cardiomyopathy

***Statement # 30:*** *Cardiovascular disease occurs in the majority of ALMS patients at some time in their lives. Cardiovascular assessment should be performed at the time of diagnosis and regularly thereafter. Standard heart failure therapy should be used in patients with heart failure and reduced ejection fraction, including angiotensin converting enzyme inhibition (ACEI) and beta-blocker titrated up to the maximum tolerated evidence-based dose, with a mineralocorticoid antagonist added thereafter. Consideration should be given to replacement of the ACEI with sacubitril valsartan in adults with an ejection fraction below 35% and persisting symptoms. Further consideration should be given to the addition of ivabradine if the heart rate is > 72/min despite optimisation, and cardiac resynchronisation therapy in those with QRS duration > 130 ms and persistent symptoms. Diuretics are recommended to reduce symptoms and signs of CHF.*

- *Level of evidence: A*
- *Strength of recommendation: 1*
- *Experts’ opinion: completely agree (92%), mostly agree (8%), partially agree (0%), mostly disagree (0%) and completely disagree (0%).*

The following cardiac evaluations should be assessed at presentation and thereafter at regular intervals:

- Standard 12-lead electrocardiograms (ECGs) – yearly
- Transthoracic echocardiograms – yearly or as per clinical need
- Cardiac magnetic resonance (CMR) imaging for older children and adult at baseline and 3 to 5 yearly intervals.

***Statement # 31***: *Although in the general population of patients with HF, a high proportion of deaths occur suddenly and unexpectedly due to electrical disturbance, the risk of arrhythmia is not well defined. Although there have been concerns raised regarding the efficacy of implantable cardioverter-defibrillators (ICD) in non-ischaemic cardiomyopathy, current standard indications should be followed, including:*

- *Implantation of an ICD in those recovering from aborted sudden cardiac death or proven ventricular tachyarrhythmia causing haemodynamic instability in those with survival expected > 1 year;*
- *Implantation of an ICD in those with advanced symptoms and reduced ejection fraction < 35% despite optimal medical therapy with survival expected > 1 year*

- *Level of evidence: B*
- *Strength of recommendation: 1*
- *Experts’ opinion: completely agree (66%), mostly agree (17%), partially agree (17%), mostly disagree (0%) and completely disagree (0%).*

***Statement # 32:*** *In those presenting with acute HF who fail to respond to standard medical therapy, consideration should be given to acute mechanical circulatory support. At present, these are used in those who have end-stage heart failure but are eligible for heart transplantation as a bridge to transplantation. Cardiac transplantation is an option in ALMS, although mechanical circulatory support has not yet been offered as an alternative destination therapy in patients.*

- *Level of evidence: C*
- *Strength of recommendation: 2*
- *Experts’ opinion: completely agree (50%), mostly agree (42%), partially agree (8%), mostly disagree (0%) and completely disagree (0%).*

### Cardiovascular risk

***Statement # 33:*** *Adult patients with ALMS are at an increased risk of premature ischemic cardiovascular disease due to their extreme IR and associated metabolic complications such as dyslipidaemia, hypertension, T2DM and NASH. Their cardiovascular risk should be modified using the current national guidelines for secondary prevention.*

- *Level of evidence: B*
- *Strength of recommendation: 1*
- *Experts’ opinion: completely agree (69%), mostly agree (31%), partially agree (0%), mostly disagree (0%) and completely disagree (0%).*

### Respiratory system

***Statement # 34:*** *Respiratory assessment should be performed at diagnosis and regularly after if there are any respiratory symptoms. A chest X-ray would be the initial investigation of choice. A restrictive pattern on Lung Function testing is often due to extrapulmonary restriction. Many patients will unfortunately find it difficult to do conventional Lung Function Testing. A low threshold for HRCT Thorax is advisable in cases of unexplained cough or breathlessness to rule out fibrotic lung disease.*

- *Level of evidence: C*
- *Strength of recommendation: 2*
- *Experts’ opinion: completely agree (70%), mostly agree (15%), partially agree (15%), mostly disagree (0%) and completely disagree (0%).*

***Statement # 35:*** *Lower respiratory tract infection in ALMS patients can sometimes progress rapidly and strategies in prevention such as a regular winter flu jab and pneumonia vaccine should be sought proactively. Patients with ALMS who contracted moderate to severe pneumonia should be treated aggressively in an intensive care set up with a particular attention of supporting their cardiorespiratory and renal functions.*

- *Level of evidence: B*
- *Strength of recommendation: 2*
- *Experts’ opinion: completely agree (79%), mostly agree (14%), partially agree (7%), mostly disagree (0%) and completely disagree (0%).*

***Statement # 36:*** *Patients with ALMS are prone to hypoxic episodes during minor surgical procedures, anaesthesia or severe infection. Care must be taken during sedation for surgical procedures. Extubation post-surgery should take place carefully after adequate oxygenation with close monitoring of oxygenation and cardiac status until 24 h post full recovery.*

- *Level of evidence: C*
- *Strength of recommendation: 2*
- *Experts’ opinion: completely agree (84%), mostly agree (8%), partially agree (8%), mostly disagree (0%) and completely disagree (0%).*

#### Endocrine and metabolism

##### Obesity and insulin resistance

***Statement # 37:*** *ALMS patients should regularly be screened for metabolic complications such as T2DM, dyslipidaemia and hypertension. A structured and personalised lifestyle programme should be encouraged and supported to promote healthy eating, regular exercise and healthy weight attainment and maintenance.*

- *Level of evidence: B*
- *Strength of recommendation: 1*
- *Experts’ opinion: completely agree (88%), mostly agree (12%), partially agree (0%), mostly disagree (0%) and completely disagree (0%).*

Almost all individuals with ALMS are prone to obesity and IR. The severity and degree of complications of IR increase with age and body habitus. Metabolic complications improve with weight loss and a structured and personalised lifestyle programme should be encouraged and supported [13, 45, 60, 62, 80].

Treatment for T2DM and obesity in ALMS include diet, weight management, and lifestyle modification counselling, provided by a physician and dietician [3, 27, 52, 62, 81]. Regular aerobic exercise with adaptations for the blind are recommended to control both obesity and diabetes [3, 27, 31, 62]. IR / T2DM should be treated as in the general population. Younger patients rarely require insulin, but some patients require long term insulin in very-high doses. Many patients with ALMS respond to insulin-sensitizing agents such as metformin and/or thiazolidinediones (TZDs) and/or dipeptidyl peptidase 4 (DPP4) inhibitors [31, 52, 62]. Sodium-Glucose Transport Protein 2 inhibitor such as empaglifozin, canaglifozin has been shown to benefit diabetic patient with chronic kidney disease (CKD) and heart disease and therefore all adult ALMS patient should be offered unless contraindicated. In case of compelling need to promote weight loss consider a Glucagon-like Peptide 1 receptor agonist.

Treatment of hypertriglyceridemia must include good metabolic control. Some patients with severe hypertriglyceridemia responded to low-fat diet combined with statins and nicotinic acid [31, 81]. Other medications, especially for severe hypertriglyceridemia, include omega-3 fatty acids and fibrates.

A clinical trial using MC4R agonist, setmelanotide, in patients with ALMS is currently underway in treating hyperphagia related weight gain.

##### NAFLD

***Statement # 38:*** *Advanced NAFLD is common in adult patients and needs careful monitoring (yearly) using liver function tests, platelet count, liver ultrasound and non-invasive tests of liver fibrosis, namely transient elastography (Fibroscan©) and Enhanced Liver Fibrosis [ELF] test.*

- *Level of evidence: B*
- *Strength of recommendation: 1*
- *Experts’ opinion: completely agree (87%), mostly agree (13%), partially agree (0%), mostly disagree (0%) and completely disagree (0%)*

Even though data are still emerging, the use of non-invasive fibrosis tests can enable a safe, repeatable assessment of advanced liver fibrosis and potentially avoid the need for liver biopsy to assess disease severity. In cases of cirrhosis, upper gastrointestinal endoscopy should be performed to assess for evidence of varices and managed as per liver guidelines (i.e. beta-blockers and/or variceal banding) [82, 83] and liver ultrasound 6-monthly to screen for hepatocellular carcinoma. Patients with complications of advanced liver disease and/or portal hypertension should be evaluated for possible early liver transplantation [53].

##### Hypothyrodism and hypogonadism

***Statement # 39:*** *Hypothyroidism is common in patients with ALMS and all individuals should have their thyroid function assessed on a yearly basis and treated accordingly.*

- *Level of evidence: B*
- *Strength of recommendation: 1*
- *Experts’ opinion: completely agree (80%), mostly agree (20%), partially agree (0%), mostly disagree (0%) and completely disagree (0%).*

***Statement # 40:*** *As children approach puberty, gonadotropin and pituitary hormones should be assessed. Male hypogonadism in adult is almost universal and should be treated with testosterone replacement as per local guidelines*

- *Level of evidence: B*
- *Strength of recommendation: 1*
- *Experts’ opinion: completely agree (79%), mostly agree (21%), partially agree (0%),mostly disagree (0%) and completely disagree (0%).*

### Kidney and bladder dysfunction

***Statement # 41:*** *Kidney disease in ALMS is common, starts early and progresses with age leading to advanced Chronic Kidney Disease at a young age. Kidney function must be monitored regularly and refer to nephrologists as needed. Patients with Advanced Kidney Disease should be evaluated for possible early kidney transplant.*

- *Level of evidence: B*
- *Strength of recommendation: 1*
- *Experts’ opinion: completely agree (80%), mostly agree (20%), partially agree (0%), mostly disagree (0%) and completely disagree (0%).*

***Statement # 42:*** *Individuals with ALMS should have their history reviewed for symptoms suggestive of neurogenic bladder (recurrent urinary tract infection, nocturia, incomplete evacuation, dribbling) and be referred for urologic evaluation if symptoms are present.*

- *Level of evidence: B*
- *Strength of recommendation: 1*
- *Experts’ opinion: completely agree (92%), mostly agree (8%), partially agree (0%), mostly disagree (0%) and completely disagree (0%).*

### Genetic counselling

***Statement # 43:*** *For genetic confirmation of the diagnosis, two ALMS1 biallelic variations have to be detected in the proband. When a pathogenic variant is detected, family segregation is recommended in order to establish the parental alleles. As a recessive model of inheritance, parents are obligate heterozygous carriers of one of the mutations detected in the proband. Heterozygotes carriers are asymptomatic and are not at risk of developing the disorder. Being, both parents, heterozygous carriers, the likelihood to have an affected child is 25%, having a carrier child 50% and being unaffected and not a carrier25%. Although it is not a very common mechanism in ALMS1 gene,* de novo *mutations should be considered. In such cases, paternity testing to confirm* de novo *mutations is highly recommended.*

- *Level of evidence: A*
- *Strength of recommendation: 1*
- *Experts’ opinion: completely agree (72%), mostly agree (21%), partially agree (7%), mostly disagree (0%) and completely disagree (0%).*

### Prenatal diagnosis

***Statement # 44:*** *Prenatal diagnosis and pre-implantation genetic diagnosis is possible when pathogenic variants have been identified in an affected family member.*

- *Level of evidence: A*
- *Strength of recommendation: 1*
- *Experts’ opinion: completely agree (80%), mostly agree (0%), partially agree (20%), mostly disagree (0%) and completely disagree (0%).*

To date, there is no information in the literature about female pregnancies or male fathering in patients with ALMS. For women who wish to become pregnant, a complete sex hormones evaluation should be required. In men, semen analysis to investigate their fertility should be suggested.

### Mental wellbeing

***Statement # 45:*** *Clinicians, caregivers and individuals with ALMS should be aware that there is an increased prevalence of behavioural problems and other psychiatric disorders such as anxiety and depression. There should be a low threshold for referral to a clinical psychology/behavioural therapy team as appropriate, and for the use of both non-pharmacological and/or pharmacological treatments.*

- *Level of evidence: C*
- *Strength of recommendation: 2*
- *Experts’ opinion: completely agree (88%), mostly agree (12%), partially agree (0%), mostly disagree (0%) and completely disagree (0%).*

The psychosocial impact of growing up and living with a disease leading to deafblindness is devastating. With support, young adult will either adapt and therefore cope well, or fail to adapt and struggle. It is even more disabling when hearing start to deteriorate. In addition, the realisation of the progressive and severe nature of the disease leads to anxiety, isolation and depression. It is paramount that patients with ALMS have regular assessment and support as required in order to maintain an optimal quality of life.

***Statement # 46****: Highly specialized Care for dual sensory loss should be considered for deaf-blind individuals with ALMS. Children deaf-blind require thoughtful and unique educational approaches in order to ensure they have the opportunity to reach their full potential. The assessment of the sensorial impairment is of high importance and will include communication evaluation, social interactions, emotional wellbeing, mobility, assistive technology, habitation and rehabilitation potential.*

- *Level of evidence: C*
- *Strength of recommendation: 2*
- *Experts’ opinion: completely agree (88%), mostly agree (12%), partially agree (0%), mostly disagree (0%) and completely disagree (0%).*

Visual cues in speech perception, speech recognition and sound localisation and distance evaluation in dual sensory loss is of particular importance [84]. A literature meta-analysis has well determined the key findings of the acquired deaf-blindness that defines the Alström group (not taking into account other manifestations of the disease) [75]:

- **For communication**: experiences of changing communication needs due to progressive sensory loss, reluctance to admit their hearing impairment with family, friends, and others;
- **For mobility:** experiences of embarrassment due to frequent bumping into objects/people; reported feeling stigmatised to use assistive devices such as canes in public and lack of mobility instructors;
- **Functioning in daily life:** experiences of more difficulty in maintaining independence; tend to need more help from others to remain independent when impairment worsens; experiences of reduced independence in shopping, food preparation, reading, house cleaning, watching television, reading books, listening to music, and use of technology;
- **Social interactions:** experiences of constant social isolation in life due to progressive impairments, on going loss of independence, and lack of the communities understanding of how to interact with deafblind people.
- **Feelings:** feelings of worthlessness, loneliness, emptiness, uncertainty, fear of losing independence, and concerns about forming relationships, being rejected by relatives and friends, and constant concern for the future; feeling depressed and suicidal thoughts

***Statement # 47:*** *All active family members should be involved in full discussion of the implications of the syndrome as appropriate for the age of the child. Whether mainstream or special schooling for the blind is chosen, a care plan including family nutrition, access to exercise, and integration in the community should be looked at.*

- *Level of evidence: C*
- *Strength of recommendation: 2*
- *Experts’ opinion: completely agree (94%), mostly agree (6%), partially agree (0%), mostly disagree (0%) and completely disagree (0%).*

Counselling is vital. One extensive review of the effects of living with a rare genetic disease on a family and the individual underlines the demanding nature of these situations and the need for family support [85]. However, counselling assessment tools are not always accessible/appropriate for deafblind people.

Autism – very difficult to diagnose in ALMS patient due to deafblindness and lack of appropriate assessment tools to reach a firm diagnosis. Useful information for Deafblind can be found in the following links:

- https://nationaldb.org/
- https://deafblind.org.uk/information-advice/deafblind-assessments/assessments-what-to-expect/
- https://deafblind.org.uk/information-advice/technology/

### Transition from childhood to adult life

***Statements # 48:*** *Most young people with ALMS are expected to reach adulthood with complex medical and psychosocial needs. The process of transition from paediatric to adult services should begin early and must include appropriate services in the community to provide a seamless transition from childhood to adult life. Individuals with ALMS may benefit from a detailed assessment identifying barriers to independence in particular the dual sensory loss.*

- *Level of evidence: B*
- *Strength of recommendation: 1*
- *Experts’ opinion: completely agree (88%), mostly agree (12%), partially agree (0%), mostly disagree (0%) and completely disagree (0%).*

### Advance care planning

***Statements # 49:*** *Specialist centre care providers, family physician/paediatrician and local palliative care services should develop close working links to support individuals and families with ALMS through the lifespan, including: a) advanced care planning with regular updating. b) proper flow of communication and clear information for patients and their families, c) a designated point of contact for each stage in their care pathway. An individual identified as being near the end- of-life may benefit from ongoing access to palliative care services including for symptom control, respite, psychological and spiritual support.*

- *Level of evidence: B*
- *Strength of recommendation: 1*
- *Experts’ opinion: completely agree (82%), mostly agree (18%), partially agree (0%), mostly disagree (0%) and completely disagree (0%).*

### Future area of research

The natural history of ALMS has evolved in the last few years with a better understanding of the syndrome and longer survival. However, there are numerous unanswered questions about the syndrome as follow:

1. What is the best way of confirming ALMS diagnosis?
2. Why the retinal dystrophy is present in almost all patients with ALMS? How to treat it?
3. Why do ALMS patient develop cardiomyopathy as infant? Why and how does it recover?
4. The onset of cardiomyopathy varies from infancy to adulthood. Does the same mechanism apply?
5. Why are patients with ALMS extremely insulin resistant disproportioned to their weight?
6. Why does NAFLD in Alström become more accelerated in progression than the general population with excess weight?
7. Is accelerated liver fibrosis caused by the metabolic complications or *ALMS1* gene alternation itself?
8. What is the mechanism driving progressive kidney disease in ALMS?
9. Why is the syndrome heterogeneous in presentation and disease progression?
10. What is the function and the structure of ALMS1 protein?
11. Is there one or more ALMS1 proteins with different tissue-specific functions?
12. Is ALMS a ciliopathy?
13. Could it be reasonable to think about proteintype-phenotype instead of genotype-phenotype correlation in ALMS?

## Concluding remarks

These guidelines are the result of an international collaboration of experts in the care of patients with ALMS and the evidence gathered to write these recommendations are the best evidence available to the experts. These guidelines address the management of children and adults affected by ALMS and are intended to facilitate optimal care to all ALMS patients worldwide. In addition, it defines standards of care against which practice can be audited and best practice can be spread.

## Data Availability

Data sharing not applicable to this article as no datasets were generated or analysed during the current study.

## Abbreviations

ACEI: Angiotensin Converting Enzyme Inhibition
AGREE II: Appraisal of Guidelines for Research & Evaluation
ALMS: Alström Syndrome
AT: Adipose Tissue
BMI: Body Mass Index
CKD: Chronic Kidney Disease
CHF: Congestive Heart Failure
CMR: Cardiac Magnetic Resonance
DPP4: Dipeptidyl Peptidase 4
DTI: Diffusion Tensor Imaging
ELF: Enhanced Liver Fibrosis
ENT: Ear, Nose and Throat
ERG: Electroretinography
FAF: Fundus Auto Fluorescence
GDG: Guidelines Development Group
GH: Growth Hormone
GLUT4: Glucose Transporter Protein Type-4
HF: Heart Failure
HRCT: High-Resolution Computed Tomography
ICD: Implantable Cardioverter-Defibrillators
IR: Insulin Resistance
KCO: Carbon Monoxide Transfer Coefficient
LV: Left Ventricular
MDT: Multi-professional team
MLPA: Multi Ligation Probe Assay
MRI: Magnetic Resonance Imaging
NAFLD: Non-alcoholic Fatty Liver Disease
NASH: Non-Alcoholic Steatohepatitis
OCT: Optic Coherence Tomography
rhGH: Recombinant Human Growth Hormone
T2DM: Type 2 Diabetes Mellitus
TZDs: Thiazolidinediones
VBM: Voxel-Based Morphometry
WES: Whole-Exome Sequencing
WGS: Whole Genomic Sequencing

## References

[1] Alström CH, Hallgren B, Nilsson LB, Asander H (1959). Retinal degeneration combined with obesity, diabetes mellitus and Neurogenous deafness: a specific syndrome (not hitherto described) distinct from the Laurence-moon-Bardet-Biedl syndrome: a clinical, Endocrinological and genetic examination based on a large pedigree. Acta Psychiatr Neurol Scand Suppl.

[2] Paisey RB, Barrett T, Carey CM, Hiwot T, Cramb R, White A (2011). Rare disorders presenting in the diabetic clinic: an example using audit of the NSCT adult Alström clinics. Pract Diab.

[3] Marshall JD, Hinman EG, Collin GB, Beck S, Cerqueira R, Maffei P (2007). Spectrum of ALMS1 variants and evaluation of genotype-phenotype correlations in Alström syndrome. Hum Mutat.

[4] Collin GB, Marshall JD, Ikeda A, So WV, Russell-Eggitt I, Maffei P (2002). Mutations in ALMS1 cause obesity, type 2 diabetes and neurosensory degeneration in Alström syndrome. Nat Genet.

[5] Goldstein JL, Fialkow PJ (1973). The Alström syndrome. Report of three cases with further delineation of the clinical, pathophysiological, and genetic aspects of the disorder. Medicine (Baltimore).

[6] Collin GB, Marshall JD, Cardon LR, Nishina PM (1997). Homozygosity mapping at Alström syndrome to chromosome 2p. Hum Mol Genet.

[7] Macari F, Lautier C, Girardet A, Dadoun F, Darmon P, Dutour A (1998). Refinement of genetic localization of the Alström syndrome on chromosome 2p12-13 by linkage analysis in a north African family. Hum Genet.

[8] Collin GB, Marshall JD, Naggert JK, Nishina PM (1999). TGFA: exon-intron structure and evaluation as a candidate gene for Alström syndrome. Clin Genet.

[9] Hearn T, Renforth GL, Spalluto C, Hanley NA, Piper K, Brickwood S (2002). Mutation of ALMS1, a large gene with a tandem repeat encoding 47 amino acids, causes Alström syndrome. Nat Genet.

[10] Collin GB, Cyr E, Bronson R, Marshall JD, Gifford EJ, Hicks W (2005). Alms1-disrupted mice recapitulate human Alström syndrome. Hum Mol Genet.

[11] Hearn T, Spalluto C, Phillips VJ, Renforth GL, Copin N, Hanley NA (2005). Subcellular localization of ALMS1 supports involvement of centrosome and basal body dysfunction in the pathogenesis of obesity, insulin resistance, and type 2 diabetes. Diabetes..

[12] Titomanlio L, De Brasi D, Buoninconti A, Sperandeo MP, Pepe A, Andria G (2004). Alström syndrome: intrafamilial phenotypic variability in sibs with a novel nonsense mutation of the ALMS1 gene. Clin Genet.

[13] Minton JA, Owen KR, Ricketts CJ, Crabtree N, Shaikh G, Ehtisham S (2006). Syndromic obesity and diabetes: changes in body composition with age and mutation analysis of ALMS1 in 12 United Kingdom kindreds with Alstrom syndrome. J Clin Endocrinol Metab.

[14] Marshall JD, Muller J, Collin GB, Milan G, Kingsmore SF, Dinwiddie D (2015). Alström syndrome: mutation spectrum of ALMS. Hum Mutat.

[15] Astuti D, Sabir A, Fulton P, Zatyka M, Williams D, Hardy C (2017). Monogenic diabetes syndromes: locus-specific databases for Alström, Wolfram, and thiamine-responsive megaloblastic anemia. Hum Mutat.

[16] Sanyoura M, Woudstra C, Halaby G, Baz P, Senée V, Guillausseau PJ (2014). A novel ALMS1 splice mutation in a non-obese juvenile-onset insulin-dependent syndromic diabetic patient. Eur J Hum Genet.

[17] Lee NC, Marshall JD, Collin GB, Naggert JK, Chien YH, Tsai WY (2009). Caloric restriction in Alström syndrome prevents hyperinsulinemia. Am J Med Genet A.

[18] Taşkesen M, Collin GB, Evsikov AV, Güzel A, Özgül RK, Marshall JD (2012). Novel Alu retrotransposon insertion leading to Alström syndrome. Hum Genet.

[19] Ozantürk A, Marshall JD, Collin GB, Düzenli S, Marshall RP, Candan Ş (2015). The phenotypic and molecular genetic spectrum of Alström syndrome in 44 Turkish kindreds and a literature review of Alström syndrome in Turkey. J Hum Genet.

[20] Malm E, Ponjavic V, Nishina PM, Naggert JK, Hinman EG, Andréasson S (2008). Full-field electroretinography and marked variability in clinical phenotype of Alström syndrome. Arch Ophthalmol.

[21] Nasser F, Weisschuh N, Maffei P, Milan G, Heller C, Zrenner E (2018). Ophthalmic features of cone-rod dystrophy caused by pathogenic variants in the ALMS1 gene. Acta Ophthalmol.

[22] Vingolo EM, Salvatore S, Grenga PL, Maffei P, Milan G, Marshall JD (2010). High-resolution spectral domain optical coherence tomography images of Alström syndrome. J Pediatr Ophthalmol Strabismus.

[23] Khan AO, Bifari IN, Bolz HJ (2015). Ophthalmic features of children not yet diagnosed with Alstrom syndrome. Ophthalmology.

[24] Dotan G, Khetan V, Marshall JD, Affel E, Armiger-George D, Naggert JK (2017). Spectral-domain optical coherence tomography findings in Alström syndrome. Ophthalmic Genet.

[25] Marshall JD, Bronson RT, Collin GB, Nordstrom AD, Maffei P, Paisey RB (2005). New Alström syndrome phenotypes based on the evaluation of 182 cases. Arch Intern Med.

[26] Marshall JD, Beck S, Maffei P, Naggert JK (2007). Alström syndrome. Eur J Hum Genet.

[27] Marshall JD, Maffei P, Collin GB, Naggert JK (2011). Alström syndrome: genetics and clinical overview. Curr Genomics.

[28] Welsh LW (2007). Alström syndrome: progressive deafness and blindness. Ann Otol Rhinol Laryngol.

[29] Izzi C, Maffei P, Milan G, Tardanico R, Foini P, Marshall JD (2011). The case: familial occurrence of retinitis Pigmentosa, deafness and RENAL involvement. Kidney Int.

[30] Bahmad F, Costa CS, Teixeira MS, Jd BF, Viana LM, Marshall J (2014). Familial Alström syndrome: a rare cause of bilateral progressive hearing loss. Braz J Otorhinolaryngol.

[31] Paisey RB, Steeds R, Barrett T, Williams D, Geberhiwot T, Gunay-Aygun M, Adam MP, Ardinger HH, Pagon RA (2003). Alström syndrome. GeneReviews®.

[32] Sebag J, Albert DM, Craft JL (1984). The Alström syndrome: ophthalmic histopathology and retinal ultrastructure. Br J Ophthalmol.

[33] Lindsey S, Brewer C, Stakhovskaya O, Kim HJ, Zalewski C, Bryant J (2017). Auditory and Otologic profile of Alström syndrome: comprehensive single center data on 38 patients. Am J Med Genet A.

[34] Edwards NC, Moody WE, Yuan M, Warfield AT, Cramb R, Paisey RB (2015). Diffuse left ventricular interstitial fibrosis is associated with sub-clinical myocardial dysfunction in Alström syndrome: an observational study. Orphanet J Rare Dis.

[35] Brofferio A, Sachdev V, Hannoush H, Marshall JD, Naggert JK, Sidenko S (2017). Characteristics of cardiomyopathy in Alström syndrome: prospective single-center data on 38 patients. Mol Genet Metab.

[36] Ponikowski P, Voors AA, Anker SD (2016). 2016 ESC guidelines for the diagnosis and treatment of acute and chronic heart failure: the task force for the diagnosis and treatment of acute and chronic heart failure of the European Society of Cardiology (ESC) developed with the special contribution of the heart failure association (HFA) of the ESC. Eur Heart J.

[37] Maisel A, Mueller C, Adams K, Anker SD, Aspromonte N, Cleland JGF (2008). State of the art: using natriuretic peptide levels in clinical practice. Eur J Heart Fail.

[38] Makaryus AN, Zubrow ME, Marshall JD, Gillam LD, Mangion JR (2007). Cardiac manifestations of Alström syndrome: echocardiographic findings. J Am Soc Echocardiogr.

[39] Toulany A, Shea S, Warren AE (2006). Doppler tissue, strain, and strain rate imaging in pediatric patients with Alström syndrome: are there regional functional abnormalities?. J Am Soc Echocardiogr.

[40] Loudon MA, Bellenger NG, Carey CM, Paisey RB (2009). Cardiac magnetic resonance imaging in Alström syndrome. Orphanet J Rare Dis.

[41] Corbetti F, Razzolini R, Bettini V, Marshall JD, Naggert J, Tona F (2013). Alström syndrome: cardiac magnetic resonance findings. Int J Cardiol.

[42] Zulato E, Favaretto F, Veronese C, Campanaro S, Marshall JD, Romano S, et al. ALMS1-deficient fibroblasts over-express extra-cellular matrix components, display cell cycle delay and are resistant to apoptosis. PLoS One. 2011;6(4):e19081. 10.1371/journal.pone.0019081 PMC3082548 21541333

[43] Jatti K, Paisey R, More R (2012). Coronary artery disease in Alström syndrome. Eur J Hum Genet.

[44] Romano S, Maffei P, Bettini V, Milan G, Favaretto F, Gardiman M (2013). Alström syndrome is associated with short stature and reduced GH reserve. Clin Endocrinol (Oxf).

[45] Han JC, Reyes-Capo DP, Liu CY, Reynolds JC, Turkbey E, Turkbey IB (2018). Comprehensive endocrine-metabolic evaluation of patients with Alström syndrome compared with BMI-matched controls. J Clin Endocrinol Metab.

[46] Mihai CM, Catrinoiu D, Toringhibel M, Stoicescu RM, Ticuta NP, Anca H (2009). Impaired IGF1-GH axis and new therapeutic options in Alström syndrome patients: a case series. Cases J.

[47] Tai TS, Lin SY, Sheu WH (2003). Metabolic effects of growth hormone therapy in an Alström syndrome patient. Horm Res.

[48] Maffei P, Boschetti M, Marshall JD, Paisey RB, Beck S, Resmini E (2007). Characterization of the IGF system in 15 patients with Alström syndrome. Clin Endocrinol (Oxf).

[49] Aynaci FM, Okten A, Mocan H, Gedik Y, Sarpkaya AO (1995). A case of Alström syndrome associated with diabetes insipidus. Clin Genet.

[50] Koç E, Bayrak G, Suher M, Ensari C, Aktas D, Ensari A (2006). Rare case of Alstrom syndrome without obesity and with short stature, diagnosed in adulthood. Nephrology (Carlton).

[51] Taşdemir S, Güzel-Ozantürk A, Marshall JD, Collin GB, Ozgül RK, Narin N (2013). Atypical presentation and a novel mutation in ALMS1: implications for clinical and molecular diagnostic strategies for Alström syndrome. Clin Genet.

[52] Paisey RB, Hodge D, Williams K (2008). Body fat distribution, serum glucose, lipid and insulin response to meals in Alström syndrome. J Hum Nutr Diet.

[53] Gathercole LL, Hazlehurst JM, Armstrong MJ, Crowley R, Boocock S, O'Reilly MW (2016). Advanced non-alcoholic fatty liver disease and adipose tissue fibrosisin patients with Alström syndrome. Liver Int.

[54] Favaretto F, Milan G, Collin GB, Marshall JD, Stasi F, Maffei P (2014). GLUT4 defects in adipose tissue are early signs of metabolic alterations in Alms1GT/GT, a mouse model for obesity and insulin resistance. PLoS One.

[55] Hung YJ, Jeng C, Pei D, Chou PI, Wu DA (2001). Alstrom syndrome in two siblings. J Formos Med Assoc.

[56] Arsov T, Silva DG, O'Bryan MK, Sainsbury A, Lee NJ, Kennedy C (2006). Fat aussie--a new Alström syndrome mouse showing a critical role for ALMS1 in obesity, diabetes, and spermatogenesis. Mol Endocrinol.

[57] Lodh S, Hostelley TL, Leitch CC, O'Hare EA, Zaghloul NA (2016). Differential effects on β-cell mass by disruption of Bardet-Biedl syndrome or Alstrom syndrome genes. Hum Mol Genet.

[58] Nesmith JE, Hostelley TL, Leitch CC, Matern MS, Sethna S, McFarland R (2019). Genomic knockout of alms1 in zebrafish recapitulates Alström syndrome and provides insight into metabolic phenotypes. Hum Mol Genet.

[59] Paisey RB, Carey CM, Bower L, Marshall J, Taylor P, Maffei P (2004). Hypertriglyceridaemia in Alström's syndrome: causes and associations in 37 cases. Clin Endocrinol (Oxf).

[60] Bettini V, Maffei P, Pagano C, Romano S, Milan G, Favaretto F (2012). The progression from obesity to type 2 diabetes in Alström syndrome. Pediatr Diabetes.

[61] Quiros-Tejeira RE, Vargas J, Ament ME (2001). Early-onset liver disease complicated with acute liver failure in Alstrom syndrome. Am J Med Genet.

[62] Paisey RB, Geberhiwot T, Waterson M, Cramb R, Steeds R, Williams K (2014). Modification of severe insulin resistant diabetes in response to lifestyle changes in Alström syndrome. Eur J Med Genet.

[63] Boerwinkle C, Marshall JD, Bryant J, Gahl WA, Olivier KN, Gunay-Aygun M (2017). Respiratory manifestations in 38 patients with Alström syndrome. Pediatr Pulmonol.

[64] Khoo EY, Risley J, Zaitoun AM, El-Sheikh M, Paisey RB, Acheson AG (2009). Alström syndrome and cecal volvulus in 2 siblings. Am J Med Sci.

[65] Baig S, Paisey RB, Dawson C, Barrett TG, Maffei P, Hodson J, et al. Defining renal phenotype in Alström syndrome. Nephrol Dial Transplant. 2018. 10.1093/ndt/gfy293. 10.1093/ndt/gfy293 30307515

[66] Waldman M, Han JC, Reyes-Capo DP, Bryant J, Carson KA, Turkbey B (2018). Alström syndrome: renal findings in correlation with obesity, insulin resistance, dyslipidemia and cardiomyopathy in 38 patients prospectively evaluated at the NIH clinical center. Mol Genet Metab.

[67] Poli L, Arroyo G, Garofalo M, Choppin De Janvry E, Intini G, Saracino A (2017). Kidney transplantation in Alström syndrome: case report. Transplant Proc.

[68] Paisey RB, Darby T, George AM, Waterson M, Hewson P, Paisey CF (2016). Prediction of protective sensory loss, neuropathy and foot ulceration in type 2 diabetes. BMJ Open Diabetes Res Care.

[69] Nelson SP (2000). Prevalence of symptoms of gastroesophageal reflux during childhood: a pediatric practice-based survey. Arch Pediatr Adolesc Med.

[70] Paisey RB, Smith J, Carey C, Barrett T, Campbell F, Maffei P (2015). Duration of diabetes predicts aortic pulse wave velocity and vascular events in Alström syndrome. J Clin Endocrinol Metab.

[71] Frölander HE, Möller C, Marshall JD, Sundqvist A, Rönnåsen B, Falkensson L (2014). Theory-of-mind in adolescents and young adults with Alström syndrome. Int J Pediatr Otorhinolaryngol.

[72] Citton V, Favaro A, Bettini V, Gabrieli J, Milan G, Greggio NA (2013). Brain involvement in Alström syndrome. Orphanet J Rare Dis.

[73] Dollfus H, Rossignol S (2019). Protocole national de diagnostic et de Soins. PNDS. Syndrome d’Alström. Haute Autorité de Santé.

[74] Florentzson R, Hallén K, Möller C (2010). Alström syndrome and cochlear implantation. The first clinical experience.

[75] Paisey RB, Leeson-Beevers K (2016). Current management of Alström syndrome and recent advances in treatment. Expert Opin Orphan Drugs.

[76] Jaiswal A, Aldersey H, Wittich W, Mirza M, Finlayson M (2018). Participation experiences of people with deafblindness or dual sensory loss: a scoping review of global deafblind literature. PLoS One.

[77] Van Groenendael S, Giacovazzi L, Davison F, Holtkemper O, Huang Z, Wang Q (2015). High quality, patient centred and coordinated care for Alstrom syndrome: a model of care for an ultra-rare disease. Orphanet J Rare Dis.

[78] Gheller F, Gallo S, Trevisi P, Caserta E, Dassie F, Maffei P, et al. Cochlear implants in Alström syndrome. Ann Otol Rhinol Laryngol. 2020. 10.1177/0003489420903061. 10.1177/0003489420903061 32019320

[79] Hill RG, Dwyer K, Tirino J, Whitley M. Cochlear implantation and mastoid obliteration in a patient with Alström syndrome. Int J Pediatr Otorhinolaryngol. 2020. 10.1016/j.ijporl.2020.109894. 10.1016/j.ijporl.2020.109894 32014736

[80] Milani D, Cerutti M, Pezzani L, Maffei P, Milan G, Esposito S (2014). Syndromic obesity: clinical implications of a correct diagnosis. Ital J Pediatr.

[81] Paisey RB (2009). New insights and therapies for the metabolic consequences of Alström syndrome. Curr Opin Lipidol.

[82] de Franchis R, Baveno VI Faculty (2015). Expanding consensus in portal hypertension: report of the BAVENO VI consensus workshop: stratifying risk and individualizing care for portal hypertension. J Hepatol.

[83] Garcia-Tsao G, Sanyal AJ, Grace ND, Carey W (2007). Practice guidelines Committee of the American Association for the study of liver diseases, practice parameters Committee of the American College of gastroenterology. Prevention and management of gastroesophageal varices and variceal hemorrhage in cirrhosis. Hepatology.

[84] Simon HJ, Levitt H (2007). Effect of dual sensory loss on auditory localization: implications for intervention. Trends Amplif.

[85] Waldboth V, Patch C, Mahrer-Imhof R, Metcalfe A (2016). Living a normal life in an extraordinary way: a systematic review investigating experiences of families of young people’s transition into adulthood when affected by a genetic and chronic childhood condition. Int J Nurs Stud.

